# Supplementary material for: Structural and Dynamic Determinants of Molecular Recognition in Bile Acid-Binding Proteins
Source: Int J Mol Sci. 2022 Jan 3;23(1):505. doi: 10.3390/ijms23010505 (PMC8745080; doi:10.3390/ijms23010505)
Supplement: Supplementary file 1 [file ijms-23-00505-s001.zip › ijms-1511050-supplementary.pdf]

SUPPLEMENTARY MATERIAL

Structural and Dynamic Determinants of Molecular Recognition in Bile Acid-Binding Proteins

Orsolya Toke

Laboratory for NMR Spectroscopy, Structural Research Centre, Research Centre for Natural Sciences, 2 Magyar tudósok körútja, H-1117 Budapest, Hungary; toke.orsolya@ttk.hu; Tel.: +36-1-382-6575

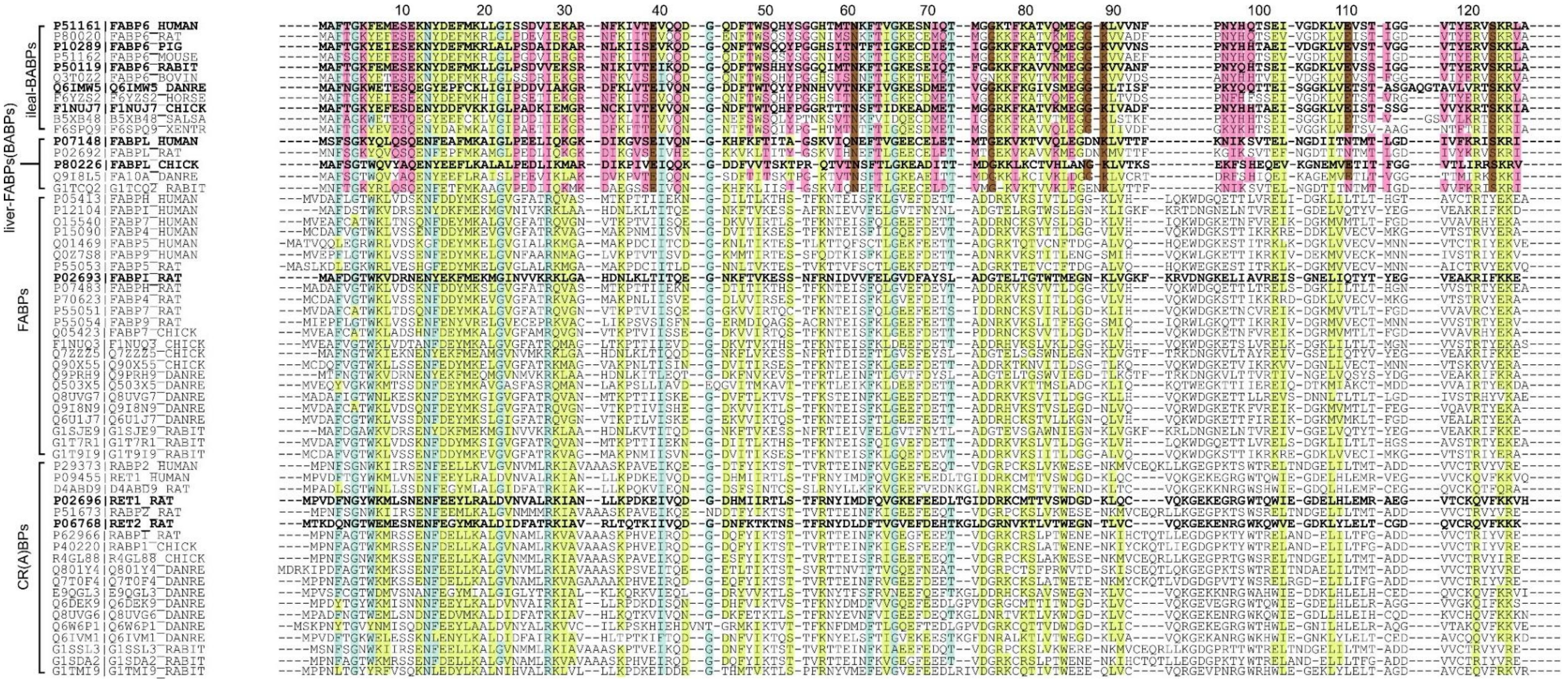

Figure S1. Sequence alignment of 59 iLBPs from subfamilies of BABPs, FABPs, and CR(A)BPs. The alignments are colored based on the Gonnet Pam250 matrix [1], using the groupings of 'STA', 'NEQK'. 'NHQK', 'NDEQ','QHRK', 'MILV', 'MILF', 'HY', 'FYW', and 'C' for the analysis of sequence similarity. Amino acid positions showing sequence identity or similarity in at least 75% of the depicted sequences are colored in blue and yellow, respectively. Additional sequence conservation observed in BABPs are colored in brown (identity) and pink (similarity). Amino acid sequence of proteins discussed in the text (BABPs, ileal: UniProt P51161, *Homo sapiens*; UniProt P10289, *Sus scrofa*; UniProt P50119, *Oryctolagus cuniculus;* UniProt *Q6IMW5, Danio rerio;* UniProt *F1NUJ7, Gallus gallus; BABPs, liver:* UniProt *P07148, Homo sapiens;* UniProt *P80226, Gallus gallus; FABPs: intestinal:* UniProt *P02693, Rattus norvegicus; CRBP-I and CRBP-II:* UniProt P02696 and UniProt P06768, respectively, *Rattus norvegicus)* are shown in bold.

1. Dayhoff, M.O.; Schwartz, R.M.; Orcutt, B.C. A model of evolutionary change in proteins. In Atlas of Protein Sequence and Structure; Dayhoff, M.O., Ed.; National Biomedical Science Foundation: Washington, DC, USA, 1978; Volume 5, Suppl. 3, pp. 345–352.
